# Estimation of Annual Secondary Lung Cancer Deaths Using Various Adjuvant Breast Radiotherapy Techniques for Early-Stage Cancers

**DOI:** 10.3389/fonc.2021.713328

**Published:** 2021-08-09

**Authors:** Jean-Philippe Pignol, Nienke Hoekstra, Derek Wilke, Hannah Dahn, Maureen Nolan, Frank Vicini

**Affiliations:** ^1^Radiation Oncology Department, Dalhousie University, Halifax, NS, Canada; ^2^Radiation Oncology Department, Erasmus MC, Rotterdam, Netherlands; ^3^Radiation Oncology, 21^st^ Century Oncology, Farmington Hills, MI, United States

**Keywords:** breast radiotherapy, secondary cancer, accelerated partial breast irradiation, brachytherapy, SBRT

## Abstract

**Purpose:**

Secondary lung cancer (SLC) can offset the benefit of adjuvant breast radiotherapy (RT), and risks compound sharply after 25 to 30 years. We hypothesized that SLC risk is mainly an issue for early-stage breast cancer, and that lives could be saved using different RT techniques.

**Patients and Methods:**

The SEER database was used to extract breast patient age, stage survival, and radiotherapy utilization over time and per stage and to assess the factors associated with increased SLC risk with a multivariable competing risk Cox model. The number of SLC was calculated using the BEIR model modified with patient survival, age, and use of RT from the SEER database. Stage distribution and number of new breast cancer cases were obtained from the NAACCR. Mean lung dose for various irradiation techniques was obtained from measurement or literature.

**Results:**

Out of the 765,697 non-metastatic breast cancers in the SEER database from 1988 to 2012, 49.8% received RT. RT significantly increased the SLC risk for longer follow-up (HR=1.58), early stage including DCIS, stage I and IIA (HR = 1.11), and younger age (HR=1.061) (all p<0.001). More advanced stages did not have significantly increased risk. In 2019, 104,743 early-stage breast patients received radiotherapy, and an estimated 3,413 will develop SLC (3.25%) leading to an excess of 2,900 deaths (2.77%). VMAT would reduce this mortality by 9.9%, hypofractionation 26 Gy in five fractions by 38.8%, a prone technique by 70.3%, 3D-CRT APBI by 43.3%, HDR brachytherapy by 71.1%, LDR by 80.7%, and robotic 4π APBI by 85.2%.

**Conclusions:**

SLC after breast RT remains a clinically significant issue for early-stage breast cancers. This mortality could be significantly reduced using a prone technique or APBI.

## Introduction

With the generalization of mammography screening, breast cancer can be diagnosed at an early stage (1), and the treatment gold standard includes breast-conserving surgery followed by whole breast radiotherapy. Radiotherapy improves the disease-free survival and local control (2–4), and there are long-term life-threatening complications that can offset the overall survival benefit. The most significant include cardiovascular morbidity and secondary cancers (3, 5).

Cardiac morbidity appears relatively soon after the radiation treatment, generally 5 to 10 years following exposure of the heart (5, 6). It is well documented in long-term reports of randomized trials or meta-analysis (4, 5). It has justified technique changes, including the generalization of breath-hold or gating techniques and the development of constraints for the mean heart dose (7, 8). Conversely, radiation-induced secondary cancers, including lung cancers, have a much delayed occurrence, compounding over time to become clinically significant after two to three decades (9–11). Using a modified version of the BEIR VII model, we previously confirmed a delay of the lifetime attributable risk (LAR) of excess lung cancers, which would be 0.33% 10 years after radiotherapy, 0.7% after 15 years, and 3% after 25 years (11).

It is difficult to use LAR in making the decision to adopt a new radiation technique, or discussing the radiation treatment with a given patient, since this risk assumes the patient would survive for a long time. It is also challenging to get a clear picture from population-based studies of the absolute number of secondary lung cancers for patients diagnosed with breast cancer today. Most meta-analysis or registry studies calculate the risk of secondary lung cancer based on patients treated a long time ago (9, 10, 12). For example, the evaluation of secondary lung cancer risk in the Inskip cohort includes 9,000 patients treated between 1935 and 1971 (10). Similarly, Grantzau meta-analysis includes patients treated between 1935 and 2007 (12). During that time frame, the utilization, techniques, and dose/fractionation of adjuvant radiotherapy have dramatically changed (7, 13). Also, early-stage breast cancer patients diagnosed today would live longer (14), meaning they are more likely to experience secondary lung cancer.

This study aimed first at, confirming that early-stage breast cancers have a higher risk of secondary lung cancer; second, comparing the true number of secondary lung cancers for different radiotherapy techniques; and third, estimating the number of lives that could be spared depending on the technique for early breast cancer patients diagnosed today.

## Materials and Method

### Patient Cohort for Probability Extraction

The SEER*Stats software version 8.3.4 was used to extract a case-listing from the Surveillance Epidemiology and End Results (SEER) 18 Custom Database with additional treatment information, which includes radiotherapy delivery information (15). This database includes reliable information on radiotherapy delivery, with a sensitivity of 68.6% and a predictive positive value of 92.2%, meaning that when radiotherapy is recorded, it was most likely delivered (16). This information was only available after 1988, so female patients with a breast cancer diagnosis after 1988 were selected. Patients with at least 5 years of follow-up and with known information on the delivery of radiotherapy were extracted. Metastatic patients were excluded, and to avoid bias, patients with a pre-existing lung cancer diagnosed before the breast one, patients who received radiotherapy for a non-breast cancer before the breast cancer, and patients who had breast cancer treated without radiotherapy and a subsequent other non-breast cancer treated with radiotherapy were excluded.

The collected information included the patient’s SEER ID, tumor site, cancer stage, age at diagnosis, month and year of diagnosis, survival in months, vital status at study cut-off, and delivery and type of radiotherapy if any. When patients had multiple breast cancer records, it is either the first one treated with radiotherapy or, if radiotherapy was not delivered, the first one diagnosed that was chosen as date of diagnosis. Patients were deemed to have received radiotherapy when the record indicated “*beam radiotherapy*”, or “*combination of beam with implanted radioisotope*”, or “*radiotherapy delivered but method not specified*”, or “*radiotherapy recommended but it is unknown if administrated*”. Conversely, patients were deemed not to have received radiotherapy when the record indicated “*no/unknown*” or “*radiotherapy recommended but refused*”. The occurrence and date of lung cancer after breast cancer was matched using the patient’s SEER ID.

The final database was stratified in prognostic groups of relatively similar sizes, including DCIS, T1a N0, T1b N0, T1c N0, Stage IIA T2 tumors, Stage IIA N1 tumors, Stage IIB, Stage IIIA, Stage IIIB, or Stage IIIC. Radiotherapy utilization was evaluated per decade for each prognostic group, ranging from 1988 to 1997, 1998 to 2007, and 2008 to 2012.

### Survival Analysis

The overall survival and lung cancer–free survival were calculated for each patient stage group using Kaplan Meyer statistics. Univariate regression analysis was used to identify the risk factors of developing secondary lung cancer. A multivariable competing risk Cox proportional hazard model was developed using a stepwise regression (PROC PHREG procedure in SAS) to account for competing risks of all-cause mortality. Independent variables included the breast cancer stage, age at diagnosis, year of diagnosis, overall survival, and the delivery of radiotherapy, as well as the resultant interaction terms between these variables. Statistical analyses were performed using SAS/STAT 14.2 (SAS Institute, Cary, NC, USA) or SPSS 25.0.0.1 (IBM Corporation, New York, NY, USA). Because multiple tests were performed, the level of significance had been set to p<0.001.

### Number of Patients at Risk of Developing a Secondary Lung Cancer

To calculate the number of patients at risk of developing a secondary cancer after breast radiotherapy, a modified BEIR VII model for a female breast cancer patient was constructed, accounting for the age and stage distribution at diagnosis, present use of radiotherapy, and survival per stage derived from the SEER cohort (11, 17). Stage distribution and number of new breast cancer was obtained from the NAACCR (18). Using this model, the risks of developing a secondary lung cancer were calculated for various mean lung dose values that were either measured in a phantom of a medium-size breast patient, or simulated using treatment planning system, or extracted from literature (11, 19–21). Techniques included whole breast irradiation (WBI) techniques excluding nodal irradiation treated in supine or prone position, delivering 42,5 Gy in 16 fractions, or 26 Gy in five fractions following the new FAST-Forward regimen (22, 23). Also, various accelerated partial breast irradiation (APBI) including 3D-conformal radiotherapy (CRT) (24, 25), volumetric modulated arc therapy (VMAT) (11), or 4π robotic radiosurgery (21) delivering lower dose in 10 or 5 fractions, high-dose rate (HDR) brachytherapy using either a balloon or multicatheter and delivering 34 Gy in 10 fractions (26), or low-dose rate 106-palladium seeds brachytherapy have been tested (27). The lung cancer mortality was derived using a 0.8 incidence-to-mortality ratio and the 2019 incidence of breast cancers in the USA (18, 28), and the number of lives that could be saved was calculated from the risk of dying of secondary lung cancer using various techniques compared to standard supine radiotherapy.

## Results

### Patients

A total of 900,085 patients with 1,079,406 cancer records were found in the SEER database. After removing stage IV, unknown stages, unknown dates of event, and patients with confounding factors, the cohort included 765,697 patients. The median age was 60 years, with an interquartile range of 21 years. Overall, 15.2% of patients had two cancer records, 1.9% had three records, and 0.24% had more than three. Breast cancer was the most frequent new cancer event recorded.

**Table 1** describes the stage distribution and median survival per stage. The median survival ranged from 22.9 years for DCIS to 6.1 years for stage IIIC. Surprisingly, there was a small number of DCIS, suggesting that *in situ* breast disease is not appropriately reported to the SEER. To compensate for this potential under-reporting, the final calculation of lung cancer risk and lives saved used the 2012–2016 North American Association of Central Cancer Registries (NAACCR) (18) stage distribution.

**Table 1:** Patient stage, age and survival distributions from the SEER 18 cohort (N=765,697).

Stage	Total (%)	Median age	Median survival
Stage 0 – DCIS	11,115 (1.45%)	59 years	22.9 years
Stage I – T1aN0	54,984 (7.18%)	61 years	22.5 years
Stage I – T1bN0	115,782 (15.1%)	63 years	20.1 years
Stage I – T1cN0	200,103 (26.1%)	62 years	19.4 years
Stage IIA – T2N0	114,818 (15.0%)	60 years	16.8 years
Stage IIA – TxN1	76,760 (10.0%)	57 years	20.2 years
Stage IIB	81,919 (10.7%)	56 years	15.3 years
Stage IIIA	58,633 (7.65%)	55 years	11.8 years
Stage IIIB	21,668 (2.83%)	62 years	4.6 years
Stage IIIC	29,915 (3.91%)	57 years	6.1 years

**Table 2** summarizes the utilization of radiotherapy per stage and over time. There was an equal split between patients receiving adjuvant radiotherapy and surgery alone, 49.8 *versus* 50.2%, respectively. The usage of radiotherapy changed over time, with a significant increase in the most recent cohorts. While there was 40.3% of patients receiving adjuvant radiotherapy during the 1988–1997 decade, this increased the following decades, 52.0% between 1998 and 2007 and 52.2% between 2008 and 2012 (p<0.001). Of note, the radiotherapy usage decreased from 65 to 53% for the very favorable T1a N0 stage group.

**Table 2:** Proportion of patients treated with radiotherapy increased over time for various breast cancer stages but for T1aN0 patients.

Stage	1988 to 1997	1998 to 2007	2008 to 2012	Relative change
DCIS	34.9% (N=3,163)	39.4% (N=6,159)	41.1% (N=1,793)	+ 17.8%
T1aN0	65.1% (N=8,654)	54.0% (N=21,346)	53.0% (N=14,372)	- 18.6%
T1bN0	49.6% (N=23,010)	59.2% (N=58,503)	57.1% (N=34,269)	+ 15.1%
T1cN0	43.1% (N=40,785)	53.3% (N=100,921)	52.1% (N=58,396)	+ 21.0%
Stage IIA – T2	30.2% (N=22,204)	41.1% (N=55,816)	41.2% (N=36,798)	+ 36.4%
Stage IIA – N1	39.1% (N=13,718)	51.9% (N=41,282)	53.8% (N=21,761)	+ 37.6%
Stage IIB	31.0% (N=13,656)	45.9% (N=41,238)	50.2% (N=27,025)	+ 61.9%
Stage IIIA	39.5% (N=12,939)	61.9% (N=29,419)	64.5% (N=16,875)	+ 63.3%
Stage IIIB	44.7% (N=4,762)	50.0% (N=11,179)	53.0% (N=5,726)	+ 18,6%
Stage IIIC	45.3% (N=7,060)	61.6% (N=14,753)	62.3% (N=8,103)	+ 37.5%

The variations for each stage are statistically significant (p < .001).

### Factors of Secondary Lung Cancers

A total of 13,689 lung cancers were detected in the cohort. Using a stepwise regression procedure, all factors were highly significantly associated (p<0.001) with an increased risk of developing secondary lung cancer, including the use of radiotherapy, an earlier cancer stage, a younger age, an earlier year of diagnosis, and a longer survival. The interaction factors were also highly significantly correlated (p<0.001). Accounting for the interaction factors, the Hazard Ratio (HR) to develop a secondary cancer was higher for patients receiving radiotherapy and treated between 1988 and 1993 (HR = 1.58, 95% CI = 1.47–1.70), compared to those receiving radiotherapy between 1994 and 1999 (HR = 1.35, 95% CI = 1.28–1.42) and those receiving radiotherapy between 2000 and 2005 (HR = 1.15, 95% CI = 1.11–1.19).

**Figure 1:**
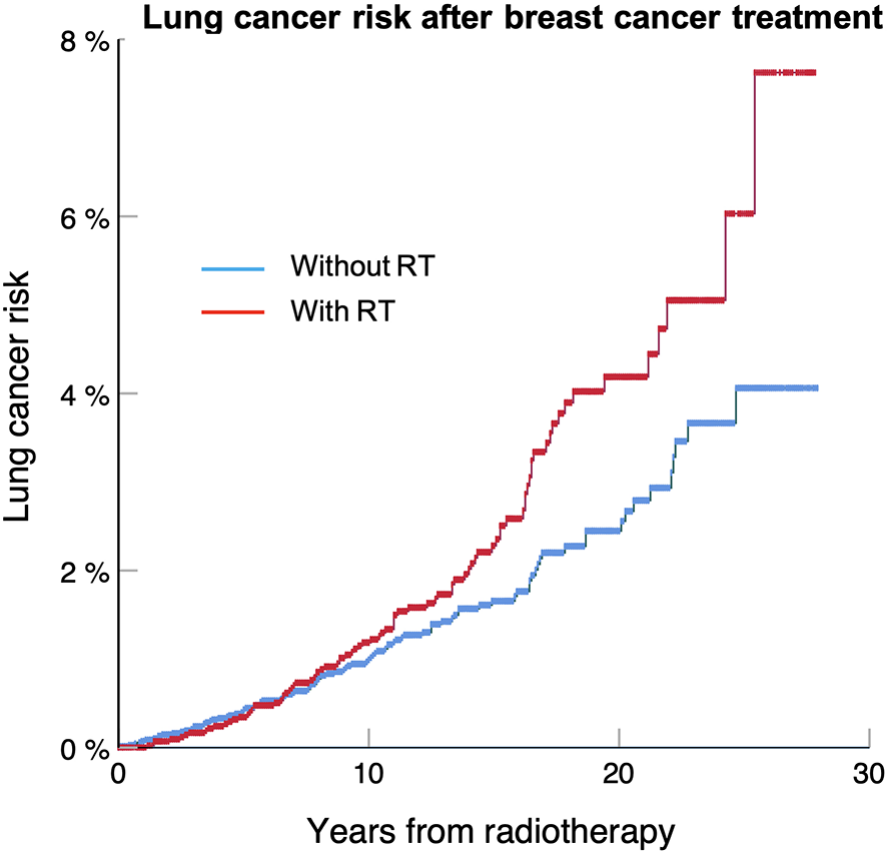
Lung cancer free survival for DCIS treated with or without radiotherapy for patients included in the SEER 18 database between 1988 and 2012. The lung cancer risk at 25 years is 8.2% for patients treated with radiotherapy compared to 4.3% without (p < 0.001).

**Table 3** shows that the risk of developing a secondary lung cancer is higher for DCIS patients, who also have the longest median of survival, with a HR of 1.66. **Figure 1** shows that the excess risk is small during the first 15 years after radiotherapy, to increase between 20 to 25 years. The risk is lower for stage I with HR about 1.2 and loses significance for Stage IIB and III, which confirms our first hypothesis that secondary lung cancer is a clinically significant issue mainly for early-stage breast cancers, and hence preventative measures should target this population.

**Table 3:** Excess risk developing a lung cancer after breast adjuvant radiotherapy compared to no radiotherapy per cancer stage.

Stage	HR	95% CI	p value
Stage DCIS	1.658	1.237 – 2.222	<0.001
Stage T1aN0	1.256	1.111 – 1.420	<0.001
Stage T1bN0	1.224	1.131 – 1.326	<0.001
Stage T1cN0	1.155	1.131 – 1.224	<0.001
Stage IIA – T2N0	1.196	1.090 – 1.312	<0.001
Stage IIA – TxN1	1.185	1.060 – 1.324	0.003
Stage IIB	0.983	0.871 – 1.110	0.78
Stage IIIA	1.050	0.904 – 1.221	0.52
Stage IIIB	1.492	1.140 – 1.953	0.004
Stage IIIC	1.084	0.851 – 1.383	0.51

### Number of Lung Cancers Using Various Breast Radiotherapy Techniques

Out of the 268,600 women diagnosed with breast cancer in the USA in 2019, an estimated 205,318 had an early stage including DCIS, stage I, and node negative stage IIA (18). Accounting for modern utilization of radiotherapy, 104,743 patients have received this treatment. We calculated that for women diagnosed with an early-stage breast cancer in 2019, a total of 3,625 of them (3.25%) will develop a radiation-induced lung cancer during their lifetime using a standard breast radiotherapy delivering a dose of 42.5 Gy in 16 fractions. Assuming a 20% lung cancer survival rate, we estimated that an excess of 2,900 patients or an absolute proportion of 2.77% of patients will die of this secondary lung cancer (**Table 4**).

**Table 4:** Annual number and mortality of secondary lung secondary cancers for patients diagnosed in 2019 with early-stage breast cancers depending on the radiotherapy technique.

Radiotherapy technique	Dose (Gy) / Fractionation	Mean lung dose	Radiation induced lung cancers	Secondary lung cancer excess death	Percent risk reduction
Standard WBI	42.5/16	2.021 Gy	3,625	2,900	–
VMAT WBI	42.5/16	1.821 Gy	3,076	2,461	9.9%
FAST-Forward WBI	26/5	1.236 Gy	2,088	1,670	38.8%
Prone WBI	42.5/16	0.600 Gy	1,076	861	70.3%
3D-CRT APBI	38.5/10	1.146 Gy	1,936	1,549	43.3%
Shorter 3D-CRT APBI	26/5	0.774 Gy	1,307	1,046	61.7%
Multicatheter HDR APBI	34/10	0.584 Gy	986	789	71.1%
Seeds LDR APBI	90/1	0.39 Gy	659	527	80.7%
Robotic 4π APBI	28.5/5	0.30 Gy	507	406	85.2%

We previously reported that using a VMAT technique optimizing the lung protection could slightly reduce the mean lung dose, eventually saving 9.9% of those excess lung cancer deaths. Using a lower total dose, 26 Gy in five fractions, would reduce the mean lung dose and eventually reduce by 38.8% the excess lung cancer mortality. Based on a literature review, Aznar reported that the prone technique enables a significant reduction of the lung exposure with a mean lung dose of about 0.6 Gy (19). This would result in the prevention of 70.3% of the excess mortality.

APBI also induces a lower mean lung dose, though not all techniques are equal. We measured for external beam 3D-CRT APBI a mean lung dose of 1.146 Gy, which would reduce the excess mortality by 43.3%, and for multicatheter HDR brachytherapy a mean lung dose of 0.584 Gy, enabling the reduction of 71.1% of the lung cancer death (11). Using low-energy 106-palladium sources, breast seed implant delivers a low dose to the lung, and 80.7% of lives could be saved (29). Taking full advantage of a 4π non-coplanar irradiation, Hoekstra reported that robotic APBI could generate the lowest mean lung dose, 0.3 Gy (21). Eventually, 85.2% of secondary lung cancers could be prevented, and more than 2,470 lives spared (**Table 4**).

## Discussion

Accounting for changes in radiation usage, protocols and techniques, current survival outcome, and patient’s stage and age characteristics, this study confirms the long-term risk of secondary lung cancer after radiotherapy for breast cancer patients diagnosed today. It adds to knowledge that the issue is statistically significant mainly for early-stage and young patients, who have a higher probability to have 20 to 30 years of survival (1, 14). This persistent risk is noticeable since several publications suggest that improved techniques reduce the risk of body exposure during radiotherapy, with, for example, a threefold reduction using field-in-field breast IMRT (30).

Although they occur late in the patient’s life, the absolute risk of excess lung cancers and deaths calculated for good prognosis cancers in the present work is high and in the same order of magnitude as radiation-induced cardiac morbidity and mortality. So, the same weight should be placed on reducing the mean lung dose when planning breast radiotherapy, as it is to minimize the mean heart dose (5). The good news is that there are several validated techniques for early-stage breast cancer which reduce the lung dose. Our study shows that they are not equal in terms of benefit. For example, VMAT only leads to a small 9.9% risk reduction. A larger benefit could be expected using a prone technique, which would reduce the mortality by as much as 70%. While the prone technique has been advocated to reduce the heart dose and to prevent skin side effects for large-breasted patients (20, 31, 32), our data suggest there is also a survival benefit.

APBI is recommended by various societies for early breast cancer stages, which our study also shows to be the main benefactors for the secondary lung cancer prevention (33–35). In 2019 two large randomized clinical trials comparing APBI to whole breast radiotherapy showed no difference at 8.6 and 10.2 years in the overall survival at the cost of a 0.7% ipsilateral breast recurrence increase (24, 25). Accounting for the survival benefit provided preventing lung cancer death, the 0.7% increase of local recurrence appears well acceptable. Importantly, our data show that the 3D-CRT technique evaluated in the RAPID and NSABP-B39 trials may not be optimal as it leads to a 43.3% reduction of the secondary lung cancer mortality. This is small compared to brachytherapy using HDR or seeds LDR with mortality reduction of, respectively, 71 and 81%. The largest benefit is calculated for the 4π robotic APBI technique with a reduction over 85%. APBI is a safe and effective substitute for whole breast radiotherapy in selected early-stage breast cancer patients. Based on the low reported toxicity (0–6.6%), APBI should be recommended in patients with life expectancies larger than 10 years.

The FAST-Forward regimen has been strongly recommended by experts to minimize travel and potential exposure of frail patients at the hospital (36, 37). As this trial shows local control and long-term side effects equivalent to standard radiotherapy over 3 weeks (23), it is likely that this regimen will be kept in the long term. However, the FAST-Forward regimen still treats the whole breast and hence has a smaller 38.8% mortality risk reduction compared to APBI techniques. To facilitate APBI adoption, single daily fraction regimens like the ACCEL trial delivering 27 Gy in five fractions (38), or the Rotterdam regimen delivering 28.5 Gy in five fractions with 4π robotic APBI, might be helpful (21).

Caution is needed in interpreting clinical outcomes from modeling, but similarly to the evaluation of cardiac morbidity, this might be the only possible strategy to assess very long-term breast radiotherapy toxicities (9). It is neither realistic nor ethical to design randomized clinical trials testing the impact of radiotherapy on the development of secondary lung cancer. It is also challenging to extract very long-term data, beyond 25 years, from population-based studies or meta-analysis because radiotherapy techniques, indications, and protocols have changed. Also, today patients are often diagnosed at an earlier stage and live longer. In the present study, which uses a very large initial cohort from the SEER database, only a small proportion of patients have very long-term follow-up: 5.1% had a follow-up exceeding 20 years, and less than 1% have follow-up between 26 and 30 years.

We used several adjustments in our model. The finding that DCIS might be underreported into the SEER database led us to opt for a hybrid model using the survival and radiotherapy utilization per stage from the SEER database, and the stage distribution from the NAACCR (18). The calculation of secondary cancer risk in various scenarios is based on the mean lung dose, which was either measured in a medium-size breast phantom or extracted from literature. The value of 2.02 Gy mean bilateral lung dose for standard breast radiotherapy is consistent with the one recently reported by Kirby for the ipsilateral lung based on a dosimetry study for 65 patients (20). It is also consistent with the values reported by Jain for the mean ipsilateral lung dose on 25 consecutive patients randomized in the NSABP B-39/RTOG 0413 protocol (39).

Caution is also needed when applying the excess lung cancer risks reported in this work to patients with different anatomy, like a smaller or a larger breast. On one hand a larger-breast patient may experience higher dose to the lung due to the larger radiation scattering volume. On the other hand, a larger body mass index means more shielding tissue and therefore less dose scattered to organs as shown on Woo’s prospective study measuring scatter dose with skin dosimeters (30). This means that the calculation of secondary lung cancer risk for a given patient should come from plan comparison testing various radiotherapy scenarios. It is possible that different anatomies may produce different classifications for the safest technique. Also, other co-factors that could have a supra-additive effect on the carcinogenic effect of radiotherapy, including smoking or the use of certain chemotherapy regimens that may have changed over time, have not been included in the model and should be considered. In a comparable modeling, Taylor used current smoker and non-smoker population mortality rates in 5-year age groups to account for the near 20-fold higher risk of lung cancers linked to tobacco consumption (9). In comparing the excess risk of secondary lung cancer for various techniques, we have used the mean lung dose as primary dose distribution metric. This was guided by the BEIR model that derives the lifetime excess risk of cancer based on the mean dose to a given target organ (40). There could be a need to add other dosimetric constraints for planning purposes. For example, the Quantec guidelines limit the volume of ipsilateral lung receiving 20 Gy or more to 30% and 30 Gy or more to 20%. To reduce the risk of pneumonitis, the volume of lung receiving 5 Gy or more is also a frequently used constraint (41). Finally, in weighing treatment options, one should carefully consider treatment safety and quality of life, and for selected early-stage breast cancer, the need for adjuvant systemic therapy may be debatable (42).

In conclusion, this study confirms that, accounting for the current utilization and techniques of radiotherapy, patient’s characteristics, and outcomes, a significant number of patients diagnosed with early breast cancer will succumb after two to three decades to radiation-induced secondary lung cancer. This could be prevented by reducing the mean lung dose with techniques like the prone technique, brachytherapy, or ultra-hypofractionated 4π robotic APBI.

## Data Availability Statement

The raw data supporting the conclusions of this article will be made available by the authors, without undue reservation.

## Author Contributions

J-PP, HD, and DW built the model and did the analysis. J-PP and NH collected experimentally the mean lung doses for the model. J-PP, NH, HD, MN, and FV wrote the first draft of the manuscript. J-PP, NH, HD, DW, MN, and FV finalized the manuscript. All authors contributed to the article and approved the submitted version.

## Conflict of Interest

Author FV is employed by 21st Century Oncology, Inc.

The remaining authors declare that the research was conducted in the absence of any commercial or financial relationships that could be construed as a potential conflict of interest.

## Publisher’s Note

All claims expressed in this article are solely those of the authors and do not necessarily represent those of their affiliated organizations, or those of the publisher, the editors and the reviewers. Any product that may be evaluated in this article, or claim that may be made by its manufacturer, is not guaranteed or endorsed by the publisher.
